# Spontaneous intracranial hypotension: features, diagnosis and management

**DOI:** 10.1007/s00415-021-10500-1

**Published:** 2021-03-08

**Authors:** Tom H. Massey, Neil P. Robertson

**Affiliations:** Division of Psychological Medicine and Clinical Neuroscience, Department of Neurology, University Hospital of Wales, Cardiff University, Heath Park, Cardiff, CF14 4XN UK

## Introduction

Spontaneous intracranial hypotension (SIH) is classically characterised by orthostatic headache and is commonly secondary to a spontaneous cerebrospinal fluid (CSF) leak near spinal nerve roots. Risk factors for SIH include connective tissue disorders, bariatric surgery and spinal pathologies such as osteophytes and disc prolapse. The annual incidence is approximately 1 in 20,000 individuals per year and the rarity of this condition has made large-scale studies of SIH difficult resulting in a limited evidence base for clinical decision-making.

This month’s journal club examines three papers reporting on the presentation, investigation and treatment of SIH. The first is a large systematic review and meta-analysis of over 2000 SIH cases. The second reports on a case series of 44 patients with SIH secondary to a rare spinal structural abnormality: cerebrospinal fluid–venous fistula. The third provides clinical information on 29 patients with frontotemporal brain-sagging syndrome, a rare, but potentially reversible, complication of SIH.

## Clinical presentation, investigation findings, and treatment outcomes of spontaneous intracranial hypotension syndrome: a systematic review and meta-analysis

This review analysed over 2000 SIH cases extracted from 144 articles up to April 30th, 2020. The majority of studies (62.5%) were retrospective, with just 14.6% being prospective. The remaining 22.9% did not clearly specify how data were collected. The diagnostic criteria for SIH varied across different studies, reflecting the lack of a universally accepted definition. Onset of symptoms was predominantly in middle age but with a large range (mean 42.5 years, range 2–88 years). Females were affected twice as often as males. Headache was the most common presenting symptom, occurring in 97% of patients. The headache was usually orthostatic (92%), and most often occipital, frontal or diffuse. A wide range of associated symptoms were reported, the most frequent of which were nausea/vomiting (54%), neck pain/stiffness (43%), hearing disturbances (28%), and dizziness (27%). Brain MRI with contrast revealed a number of characteristic abnormalities including diffuse pachymeningeal enhancement (73%), venous engorgement (57%), brain sagging (43%) and subdural collections (35%). Of note, 19% of cases had normal brain MRI.

Although the study reported that extradural CSF was detectable on spinal imaging in 48–76% of cases, it was often difficult for the exact leak site to be located. Imaging techniques such as digital subtraction myelography were most successful at locating leaks, and these were most commonly found in the thoracic spine (41%), although also reported in the cervical and lumbar regions, or at multiple levels (24%). Lumbar puncture, when performed, revealed a low opening pressure (< 60 mm CSF) in 67% of cases, with 32% having normal pressure (60–200 mm CSF). Conservative treatment, usually consisting of a long period of bed rest and hydration (up to 9 weeks), led to resolution of symptoms in 28%. If conservative measures failed, second-line therapy was usually an epidural blood patch (EBP) which was successful in 64% of cases. Larger volume EBPs (> 20 ml) were more effective than smaller volume ones (77% and 66% successful, respectively), but there was no significant difference between targeted and non-targeted EBPs. This study reported only small numbers of surgical interventions and was unable to draw detailed conclusions about their efficacy.

**Comment:** This large meta-analysis shows that in most cases of SIH, orthostatic headache and brain MRI findings are diagnostic. However, there are atypical headache presentations and almost a fifth have normal MRI brain imaging. Lumbar puncture can show low CSF pressure, but is often not required diagnostically and may worsen symptoms. Spinal imaging often cannot identify a CSF leak site and non-targeted EBP is as effective as targeted EBP. Limitations of the studies presented include that they were mostly retrospective, based on variable diagnostic criteria, and heterogeneous in their designs.

*D’Antona, L. et al. (2021) JAMA Neurol *https://doi.org/10.1001/jamaneurol.2020.4799

## Headache due to spontaneous spinal cerebrospinal fluid leak secondary to cerebrospinal fluid–venous fistula: case series

One rare cause of SIH is CSF leakage secondary to a cerebrospinal fluid–venous fistula. Such fistulae can be iatrogenic, for example after lumbar puncture or spinal surgery, but can also arise from spontaneous rupture of a perineural cyst into an adjacent vein, usually near a spinal nerve root. CSF–venous fistulae are most common in the thoracolumbar region, with 68% found between T7 and T12.

This retrospective study identified 44 patients with CSF–venous fistula at a single tertiary centre between 1994 and 2019. Females were affected more commonly than males (approximately 2:1) and symptom onset was in middle age (mean onset 52.6 years). Over 95% of patients presented with headache, often with a preceding period of episodic or chronic headaches. Interestingly, the Valsalva manoeuvre induced or exacerbated the presenting headache in 88% of cases whereas only 69% reported an orthostatic component. Brain MRI with contrast was abnormal in over 80% but CSF opening pressure, when measured, was commonly normal (7–20 mm CSF in 84%). Digital subtraction or positive pressure myelography were best at identifying fistulae, with conventional CT and MRI myelograms having a poor yield. In contrast with other CSF leaks, extradural CSF is not found on imaging; instead, myelographic contrast can be seen in an epidural vein.

Most fistulae in this series were thoracic with 76.7% being right sided for unknown reasons. 40 patients received at least one EBP, but the response was far less effective than for SIH in general: only 10% had a good response lasting at least 3 months. 42 patients had surgical treatment of their CSF–venous fistula, an average of 3 years after onset of symptoms. Surgery usually involved ligation of the involved thoracic nerve root and its associated veins, although the nerve root was preserved if the fistula was at the C7/T1 level. There were few post-operative problems reported—transient rebound intracranial hypertension occurred in 7% and one patient reported new mid-thoracic paraspinal pain. Over 80% of patients treated surgically reported improvement of symptoms, with nearly 50% achieving headache-freedom. In most cases of clinical improvement, the MRI brain abnormalities resolved.

**Comment:** This study highlights CSF–venous fistula as a rare but surgically treatable cause of SIH. In a patient with SIH who is not responding to conservative treatment or EBPs, detailed spinal imaging with contrast could be of benefit. Future prospective studies using advanced imaging techniques will likely lead to increasing diagnosis and understanding of this rare abnormality.

*Duvall, J.R. et al. (2019) Cephalalgia 39: 1847–1854*

## Behavioural variant frontotemporal dementia as a serious complication of spontaneous intracranial hypotension

A spinal CSF leak can induce low CSF pressure and sagging of the brain and brainstem. In rare cases, this can cause a clinical syndrome indistinguishable from behavioural variant frontotemporal dementia (bvFTD), consisting of inappropriate social functioning, altered personality, apathy, hyperorality and cognitive impairment. However, in contrast to bvFTD, there is no frontotemporal atrophy on MRI imaging and the clinical abnormalities are reversible on treatment of the CSF leak.

This study reports a case series of 29 patients (21 male) meeting diagnostic criteria for SIH and bvFTD at a quaternary SIH centre between 2001 and 2017. Mean age at onset of SIH was 50.6 years. The most common clinical scenario was onset of orthostatic headache followed by bvFTD symptoms an average of 2 years later. Of note, all patients reported daytime hypersomnolence and this mostly coincided with bvFTD symptoms, of which disinhibited behaviour and apathy were most prevalent. MMSE scores had a mean of 22.4/30, indicating cognitive impairment. Interestingly, the bvFTD-like symptoms were often worse towards the end of the day and seemed to have an orthostatic component.

MRI brain showed severe brain sagging, involving the midbrain and brainstem, and bilateral temporal lobe herniation in all patients. Pachymeningeal contrast enhancement was common. Extensive spinal imaging was unable to detect a CSF leak in any patient although spinal meningeal diverticula were found in 52%. Opening CSF pressure, when measured, was low (< 60 mm) in approximately half of the patients. Improvement after EBP was noticed in 86% but this was often not sustained, and most patients required further EBP(s) and/or a range of surgical interventions including spinal meningeal diverticular repair. Despite these difficulties with treatment, a good outcome of no/mild disability was reported in 72% (2-year mean follow-up time after last treatment).

**Comment:** The combination of orthostatic headache, hypersomnolence and bvFTD-like symptoms should prompt a clinician to consider brain sagging secondary to SIH. Brain MRI can confirm the diagnosis. It is important to recognise this diagnosis as symptoms are often reversible with EBPs and surgical interventions.

*Schievink, W.I. et al. (2018) Operative Neurosurgery 15: 505–515*

## Conclusion

SIH is a rare but debilitating condition, most often caused by CSF leaks near the thoracic nerve roots. Large volume non-targeted EBPs are the most effective intervention reported. In a few cases, SIH is caused by a CSF–venous fistula which requires specific surgical treatment. One rare downstream effect of prolonged SIH is brain sagging: a potentially reversible cause of bvFTD-like symptoms.

